# Predictors of transition from paliperidone palmitate 1 and 3 months (PP1M & PPP3M) to paliperidone palmitate 6 months (PP6M)

**DOI:** 10.1192/j.eurpsy.2023.942

**Published:** 2023-07-19

**Authors:** P. J. Escobedo-Aedo, J. Merayo-Cano, S. Sánchez Alonso, S. Ovejero, L. Muñoz Lorenzo, L. Mata Iturralde

**Affiliations:** 1 Psychiatry, Hospital de Torrejón, Torrejón de Ardoz; 2 Psychiatry, Fundación Jiménez Díaz, Madrid, Spain

## Abstract

**Introduction:**

Schizophrenia is a severe, chronic, mental disease. Its stability relies upon a multidisciplinary treatment, where pharmacological treatment is a key aspect. Long-acting injectable antipsychotics (LAIs) have proved efficacy in improving adherence, reducing hospitalizations and relapses, compared with oral treatment[1,2]. Paliperidone palmitate is a long-acting antipsychotic, approved by FDA in 2009 for acute and chronic treatment in schizophrenia. To date, long evidence exists regarding treatment efficacy of paliperidone palmitate 1 month (PP1M) and paliperidone palmitate 3 month (PP3M)[3]. In September 2021 a new long-acting medication was approved for schizophrenia treatment, that is, paliperidone palmitate 6 months (PP6M). This is the first LAI with 6 months duration of treatment, which means, only 2 administrations per year.

We here analyzed the factors explaining transitioning from PP1M and PP3M to PP6M treatment in a population previously described somewhere else[4].

**Objectives:**

To identify the variables explaining the transition from other long-acting formulations (PP1M and PP3M) to the new biannual formulation (PP6M) in our clinical practice.

**Methods:**

123 patients, previously diagnosed with psychotic disorders, in follow-up in our clinical center Fundación Jiménez Díaz Hospital, was analyzed. Sociodemographic factors and clinical evolution were compared in order to identify factors predicting transitioning from PP1M and PP3M to PP6M.

**Results:**

In the PP1M group, patients transitioning to PP6M had more than 6 years of evolution of disease ans active consummation of drugs, compared with patients who stayed on PP1M. Other sociodemographic were similar in both groups. Only 1 patient was readmitted in hospital since transition to PP6M and no emergency visits were accounted for people transitioned.

In the PP3M group, the majority of people transitioning to PP6M were under polypharmacy of which, 42% were on clozapine treatment. The percentage of people with schizophrenia diagnosis was significantly less than in the no transitioning group, though it remained the principal diagnosis. No other significant difference was found with regard to sociodemographic variables. Additionally, no emergency visits nor readmissions to hospital were accounted in this group.

Finally, the PP3M transitioned to PP6M significantly more than PP1M group. Although no clear variable explained this situation.

**Conclusions:**

With these results, we conclude that chronicity and drugs consummation were the main variables explaining transitioning from PP1M to PP6M. In the other hand, the main variable explaining transitioning from PP3M to PP6M was polypharmacy.

These results are preliminary and, therefore, should be taken cautiously. We will probably dilucidated future tendency in these treatment use in the upcoming months.

**Disclosure of Interest:**

None Declared

